# Engineering of *Helicobacter pylori* L-Asparaginase: Characterization of Two Functionally Distinct Groups of Mutants

**DOI:** 10.1371/journal.pone.0117025

**Published:** 2015-02-09

**Authors:** Maristella Maggi, Laurent R. Chiarelli, Giovanna Valentini, Claudia Scotti

**Affiliations:** 1 Department of Molecular Medicine, Unit of Immunology and General Pathology, University of Pavia, Pavia, Italy; 2 Department of Biology and Biotechnologies “Lazzaro Spallanzani”, Laboratory of Molecular Microbiology, University of Pavia, Pavia, Italy; 3 Department of Biology and Biotechnologies “Lazzaro Spallanzani”, Laboratory of Protein Biochemistry, University of Pavia, Pavia, Italy; Monash University, AUSTRALIA

## Abstract

Bacterial L-asparaginases have been used as anti-cancer drugs for over 4 decades though presenting, along with their therapeutic efficacy, several side effects due to their bacterial origin and, seemingly, to their secondary glutaminase activity. *Helicobacter pylori* type II L-asparaginase possesses interesting features, among which a reduced catalytic efficiency for L-GLN, compared to the drugs presently used in therapy. In the present study, we describe some enzyme variants with catalytic and *in vitro* cytotoxic activities different from the wild type enzyme. Particularly, replacements on catalytic threonines (T16D and T95E) deplete the enzyme of both its catalytic activities, once more underlining the essential role of such residues. One serendipitous mutant, M121C/T169M, had a preserved efficiency *vs* L-asparagine but was completely unable to carry out L-glutamine hydrolysis. Interestingly, this variant did not exert any cytotoxic effect on HL-60 cells. The M121C and T169M single mutants had reduced catalytic activities (nearly 2.5- to 4-fold *vs* wild type enzyme, respectively). Mutant Q63E, endowed with a similar catalytic efficiency versus asparagine and halved glutaminase efficiency with respect to the wild type enzyme, was able to exert a cytotoxic effect comparable to, or higher than, the one of the wild type enzyme when similar asparaginase units were used. These findings may be relevant to determine the role of glutaminase activity of L-asparaginase in the anti-proliferative effect of the drug and to shed light on how to engineer the best asparaginase/glutaminase combination for an ever improved, patients-tailored therapy.

## Introduction

L-asparaginases (L-ASNase) are amidohydrolases (EC 3.5.1.1) that primarily catalyze L-asparagine (L-ASN) deamination leading to the formation of L-aspartate (L-ASP) and ammonia. L-ASNases also exhibit the capability of carrying out L-glutamine (L-GLN) hydrolysis, although the efficiency toward this substrate greatly depends on the enzyme source [1]. On the basis of their affinity toward L-ASN, cellular localization and glutaminolytic activity, bacterial L-ASNases are divided into two groups. Type I L-ASNases are cytoplasmatic enzymes, constitutively expressed, present low affinity *vs* L-ASN (in the order of mM) and are also active toward L-GLN. Type II L-ASNases are periplasmic, have anaerobiosis-dependent expression, display high affinity *vs* L-ASN (in the order of μM) and low to negligible activity toward L-GLN [2].

For over 4 decades *Echerichia coli* and *Erwinia chrysantemi* type II L-ASNases (EcAII and ErA, respectively) have been used in the treatment of acute lymphoblastic leukaemia (ALL), above all in childhood [3, 4, 5, 6]. Their therapeutic efficacy can be primarily attributed to the reduction of serum L-ASN, which is essential for survival of L-asparagine synthetase (ASNS) knock-out (or down regulated) lymphoblastic tumoral cells [7, 8]. Systemic depletion of extracellular L-ASN prevents protein biosynthesis in malignant lymphoblasts which ultimately can result in tumor cell cycle inhibition or apoptosis [9].

Among the cytotoxic agents employed in regimens for acute lymphoblastic leukemia (ALL), L-ASNase II is one of the most effective and with relatively mild side effects [10]. Nevertheless, some of the latter, such as hypersensitivity, coagulative disorders, impaired liver function, pancreatitis and neurological dysfunctions hamper their clinical application [11]. Apart from hypersensitivity, all other side effects are most likely linked to the secondary L-glutaminase (L-GLNase) activity of the enzyme [12, 13, 14, 15]. In fact, massive depletion of L-GLN, the major transport form of amino nitrogen in the blood and donor of amino group for many biosynthetic reactions, can be responsible for loss of cellular functions. Several studies, however, show that this activity participates in inducing the cytotoxic effect of L-ASNase, most of all in ASNS-positive tumoral cells [12, 16, 17]. This suggests that leukemia cells environment and their genetic profile must be considered in order to obtain an effective treatment strategy [18] and that enzymes with variable activity and affinity toward L-GLN are expected to become more and more useful to devise finely patient-tailored treatments [19].

*Helicobacter pylori* CCUG 17874 type II L-asparaginase (HpASNase) displays a strong preference for L-ASN over L-GLN, good heat stability, and, most of all, high cytotoxicity [20, 21]. Interestingly, the higher S_0.5_ toward L-GLN exhibited by HpASNase with respect to the enzymes used as drugs from *E. coli* and *E. chrisantemi* could help to reduce the side effect manifestations linked to excessive L-GLN depletion in blood, especially when L-GLNase activity is not essential [19]. In general, bacterial L-ASNases exhibit a hyperbolic response toward substrates, except two enzymes [2, 22, 23], *E. coli* type I and HpASNase, which exhibit cooperativity *vs* L-ASN [23] and L-GLN [21], respectively. The basis of these enzymatic features are still to be clarified and must be searched in the structure of the molecule.

Bacterial L-asparaginases are 140–150 kDa homotetramers more accurately described as dimers of intimate dimers, each defined by A/C and B/D monomeric interactions. Each monomer is organized into two well distinct domains, a large N-terminal domain and a smaller C-terminal domain, connected by a linker region [24]. Four independent catalytic sites are located at the intersubunits interface of the intimate dimers with amino acid residues contributed by both monomers [24, 25].

The precise reaction mechanism of bacterial L-ASNses is still unknown, although it has been compared to the two-step ping-pong mechanism of classic serine proteases [25], with the involvement of two catalytic triads (Thr12-Tyr25-Glu283, and Thr89-Asp90-Lys162, respectively, in *E. coli* type II L-ASNase), and the formation of an acylenzyme as intermediate. Moreover, it implies the motion of the so-called flexible loop (EcAII residues 13–40) for correct arrangement of the active site and substrate allocation [24, 25, 26].

In order to explore the role of specific residues in the shaping of the catalytic site, site-directed mutants were expressed in recombinant form and characterized from the biochemical point of view. The most relevant ones were also tested for cell cytotoxicity and a serendipitous, double mutant was helpful in defining a critical region able to modulate GLNase activity.

## Materials and Methods

### Rationale for site direct mutagenesis

The rational engineering of HpASNase was performed starting from structural and catalytic data available from other bacterial L-ASNases, mainly from the crystallographic structure of a *H. pylori* L-ASNase (HpA, PDB ID: 2WT4, 2WLT [25]) which displays 92% of sequence identity with *H. pylori* strain CCUG 178174. Two groups of mutants, namely, catalytic triads mutants (T16D, T95E) and substrates binding and stabilization mutants (Q63E, M121C/T169M, M121C, T169M), were planned (Table 1).

**Table 1:** Site directed mutants.

**Residue number**	**Original amino acid**	**Amino acid replacement**	**Role in the wild type enzyme**	**Aim**
**16**	T	D	Catalysis and substrates binding	Active site study
**63**	Q	E	Substrate accommodation	Increase affinity to L-ASN; reduction of L-GLNase activity
**95**	T	E	Catalysis and substrates binding	Active site study
**121**	M	C	L-GLN binding	Removal of L-GLNase activity
**169**	T	M	Unclear, maybe involved in intersubunits interactions	Random mutation*

* Note: this is a serendipitous mutation introduced by PCR during the generation of the M121C mutant. Both the M121C/T169M double mutant and the single mutants were characterized.

### Generation, expression and purification of HpASNase mutants

In order to obtain enzymatic variants of *H. pylori* CCUG 17874 L-ASNase, the pDC1 plasmid, a derivative of pET101 D-TOPO (Invitrogen) containing the *ansB* wild type gene [21] was subjected to site direct mutagenesis using Quick Change XL Site-directed Mutagenesis Kit (Stratagene).

The sense and antisense mutagenic oligonucleotides used are reported in Table 2. All the mutants were checked by insert sequencing. *E. coli* BL21(DE3) pLysS cells transformed with pDC1 derivatives were grown in a ZYP-5052 medium [27] containing 100 μg/ml ampicillin and protein expression performed by auto-induction. For enzyme purification, cells were collected by centrifugation from a 1 l culture, resuspended in 50 mM Na-phosphate pH 8.0, 300 mM NaCl, 10 mM imidazole (ImOH) (buffer A), added with 1 mM PMSF, sonicated and centrifuged (8000 g, 20 min, 4°C). After an additional centrifugation at 150,000 g (30 min, 4°C), the supernatant was applied to a HisTrap 5 ml column (GE Healthcare), equilibrated in buffer A, and protein elution was performed by an ImOH discontinuous gradient (25, 50, 100, 250, 500 mM). HpASNase eluted at 250 mM ImOH. The fractions containing HpASNase were pooled together, concentrated and applied onto a HiLoad-Superdex S200 16/60 column (GE Healthcare) equilibrated in Na-phosphate 50 mM, pH 7.5 (buffer B). After elution, the fractions were checked by 12% SDS-PAGE and those containing a single band of 37 kDa were pooled together. Protein concentration was determined according to Lowry et al. [28].

**Table 2:** Sense and antisense oligonucleotides used for site direct mutagenesis.

**Mutations**		**Oligonucleotides**
**T16E**	Sense	5′-GACAGGGGGG**GA**GATTGCAGGGAGTG-3′
	Anti-sense	5′-CTGTCCCCCC**CT**CTAACGTCCCTCAC-3′
**Q63E**	Sense	5′-GGTTTCTAATATCGGCTCA**G**AAGACATGAATGAAG-3′
	Anti-sense	5′-CCAAAGATTATAGCCGAGT**C**TTCTGTACTTACTTC-3′
**T95E**	Sense	5′-GTCATCACGCATGGC**GAT**GACACTTTAGAAGAG-3′
	Anti-sense	5′-CAGTAGTGCGTACCG**CTA**CTGTGAAATCTTCTC-3′
**M121C**	Sense	5′-CTAGTGGGAGCG**TGT**CGTAACGCTGCTTC-3′
	Anti-sense	5′-GAAGCAGCGTTACG**ACA**CGCTCCCACTAG-3′
**T169M**	Sense	5′-GAGAGGTAGTTAAAA**T**GCACACCACCCACAC-3′
	Anti-sense	5′-GTGTGGGTGGTGTGC**A**TTTTAACTACCTCTC-3′

The underlined and bold letters indicate the mutated bases.

### Circular dichromism

Far-UV circular dichroism (CD) measurements were carried out using a Jasco J-700 spectropolarimeter (Jasco-Europe, Cremella, Italia) with a 1 mm path cell. Scans were performed between 190 and 250 nm at a speed of 20 nm min^-1^ with a spectral band of 2 nm and a sensitivity of 20 millidegrees. Measurements were performed in 10 mM Na-phosphate pH 7.5 at 25°C. The protein concentration was 5 μM. The Dichroweb platform (http://dichroweb.cryst.bbk.ac.uk/html/home.shtml) was used for data analysis obtained from at least 10 different scans.

### Activity assays and kinetic analyses

Both L-ASNase and L-GLNase activities were determined at 37°C with L-ASN or L-GLN, respectively, as a substrate, by the L-glutamic dehydrogenase-coupled spectrophotometric assay [29], in the same conditions adopted for the wild type enzyme [21]. Briefly, the standard reaction mixture contained 50 mM 4-(2-hydroxyethyl)piperazine-1-ethanesulfonic acid (HEPES) buffer, pH 7.5, 1 mM α-ketoglutarate (α-KG), 0.24 mM NADH, 20 U L-glutamic dehydrogenase and 5 mM L-ASN or 150 mM L-GLN, in a final volume of 0.5 ml. The reaction was started by adding the enzyme solution (5–10 μg) and NADH consumption was monitored at 340 nm with a Jasco V-550 UV/VIS spectrophotometer (Jasco-Europe).

For steady-state kinetic studies, enzyme activity was assayed in triplicate under the above conditions using at least 10 different L-ASN or L-GLN concentrations (0–10 mM L-ASN or 0–400 mM L-GLN). The reaction was started adding the substrate to the reaction mixture containing 5–15 μg enzyme. The kinetic parameters V_max_ and K_m_ were determined by Lineweaver-Burk plot, while S_0.5_ and n_H_ values were determined by Hill plot using, in all cases, the Enzyme Kinetic Module 1.1 for Sigma Plot (SPSS Inc.).

K_cat_, or turn-over number, is the number of catalytic events per second. K_m_ is the substrate concentration at which the reaction rate is half maximal. S_0.5_ is the K_m_ equivalent value for a cooperative enzyme. k_cat_/K_m_ is an index of enzyme catalytic efficiency at sub-saturating substrate concentrations. Hill coefficient (n_H_) is an empirical parameter related to the degree of cooperativity; values larger than unity indicate positive cooperativity among ligand binding sites.

### Thermal stability assay

T_50_ is the temperature at which an enzyme loses 50% of its activity in a given period of time. To evaluate the T_50_ of HpASNase variants, enzyme solutions (0.5 mg ml^-1^) were incubated at different temperatures for 10 min in 50 mM Na-phosphate, pH 7.5. After incubation, the enzyme solutions were chilled on ice and assayed for activity. The residual activity was expressed as the percentage of activity of heat-treated enzyme with respect to the untreated one.

### Cell culture and viability assay

Human promyeloblast cells (HL-60) were grown at 37°C in RPMI-1640 medium containing 100 IU ml^-1^ penicillin and 100 μg ml^-1^ streptomycin and added with 10% FBS and 2 mM L-GLN in a 5% CO_2_ humidified atmosphere. For the viability tests, 50000 cells/well were seeded in a 96-well plate and incubated at 37°C for 24 h in the presence of different concentrations (0.03–3.3- U ml^-1^) of purified recombinant *H. pylori* wild type, T16D, Q63E and M121C/T169M L-ASNase variants in phosphate buffered saline (Sigma Aldrich). As for the wild type and Q63E variants, the viability *vs* L-GLNase activity (0.03–3.8 U ml^-1^) was also determined. Each treatment was performed at least three times in different days and in triplicate wells for each condition.

Cell survival was determined by 3-(4,5-dimethylthiazol-2-yl)-2,5-diphenyltetrazolium bromide (MTT) reduction assay. The viability value was expressed as a percentage of living cells *vs* non treated sample and the values obtained at different enzyme concentrations were compared by ANOVA and Tukey test. A p-value<0.05 was considered significant. For the dose-response analysis as EC_50_ value, the data were plotted using the four parameter logistic function. The EC_50_ value indicates L-ASNase concentration inducing 50% of the maximal cytotoxic effect.

## Results

### Cloning, expression and purification of HpASNase mutants

Four mutants (T16D, T95E, Q63E, M121C) of HpASNase were initially designed and generated starting from the cloned *ansB* gene by site-direct mutagenesis. In the case of the insert planned to encode the M121C mutant, an additional PCR-derived mutation at nucleotide position 506 C>T lead to a T169M substitution in the protein molecule. After a preliminary biochemical characterization of the resulting M121C/T169M enzyme, the T169M and M121C variants were also generated.

**Figure 1:**
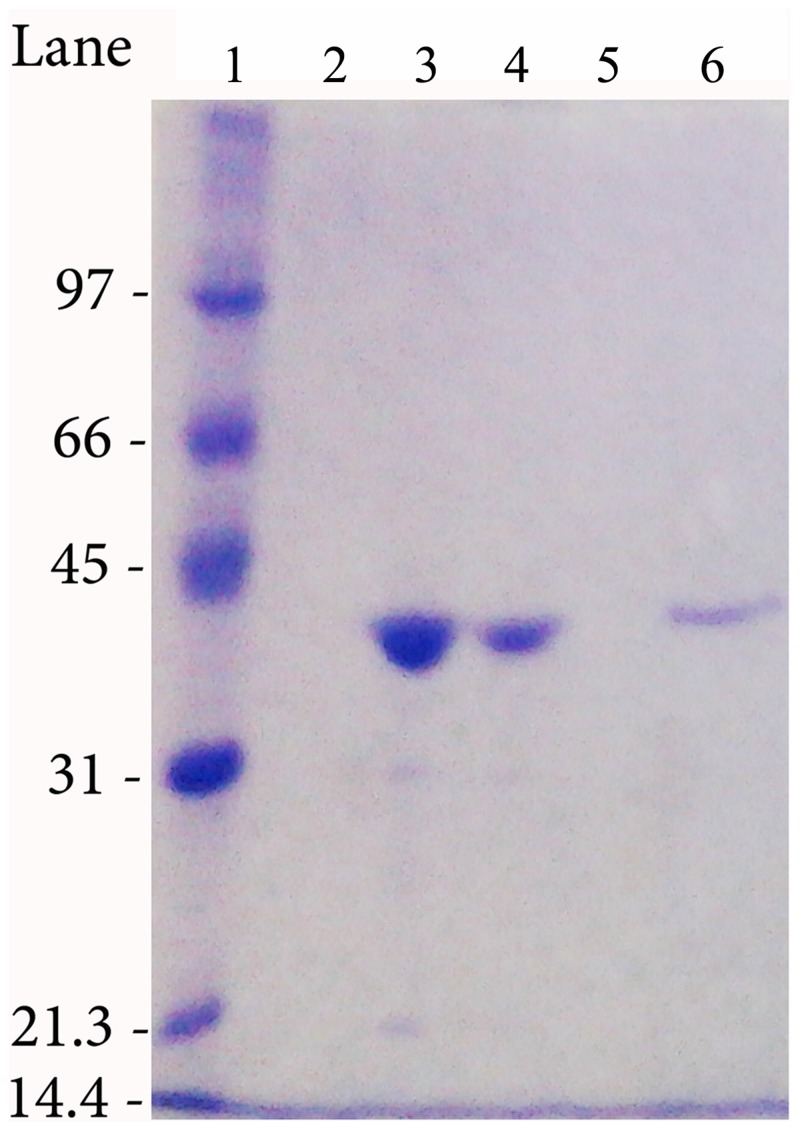
Example of SDS-PAGE of the purified HpASNase double mutant. The enzyme was run in parallel with molecular mass standards on a 12% gel and stained with Coomassie Blue R-250. Lane 1: molecular mass markers (97, 66, 45, 31, 21.3 and 14.4 kDa). Lanes 2 and 5: empty lanes. Lane 3 and 4: 250 mM ImOH Nichel fractions. Lane 6: gel filtration, pooled fractions.

All the mutants (T16D, T95E, Q63E, M121C, T169M and M121C/ T169M) were expressed in *E. coli* BL21(DE3) pLysS cells and purified to homogeneity (Fig. 1) following the procedure reported in the Material and Methods section.

### Kinetic properties

**Figure 2:**
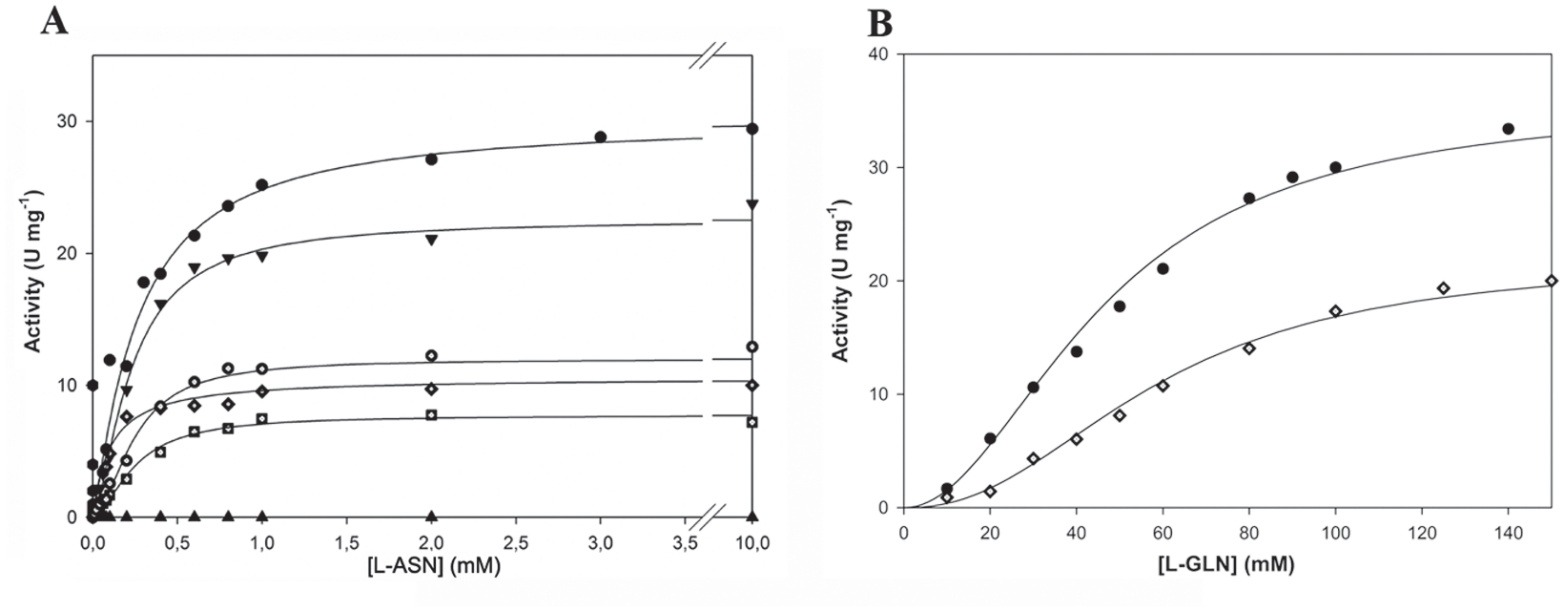
Activity dependence on substrate concentrations. Steady state kinetics of recombinant HpASNase wild type and variants as a function of L-ASN (panel A) and as a function of L-GLN (panel B). All experiments were performed at 37°C as reported in the Materials and Methods section. Symbols: black circle: wild type; black triangle pointing up: T16D; black square: T95E; diamond: Q63E; empty square: M121C; empty circle: T169M; black triangle pointing down: M121C/T169M (double mutant).

The mutants were subjected to kinetic analysis using either L-ASN or L-GLN as a substrate. At least ten different concentrations of L-ASN or L-GLN were used for each kinetics (Fig. 2A and B).

All of them, except T16D and T95E, resulted to be active toward L-ASN, although to a different extent (Fig. 2A and Table 3). Circular dichroism and analytical gel filtration experiments indicated that also these inactive mutants were properly folded and correctly arranged as tetramers (data not shown).

**Table 3:** Apparent kinetic constants of HpASNase wild type and mutant forms.

	**L-asparagine**					**L-glutamine**				
	**V_max_ (U mg^-1^)**	**k_cat_ (s^-1^)**	**S_0.5_ (mM)**	**k_cat_/S_0.5_ (s^-1^ mM^-1^)**	**n_H_**	**V_max_ (U mg^-1^)**	**k_cat_ (s^-1^)**	**S_0.5_ (mM)**	**k_cat_/S_0.5_ (s^-1^ mM^-1^)**	**n_H_**
wild type	31.22±0.90	19.26±0.56	0.29±0.03	66.4	1.0±0.06	35.8±2.25	22.10±1.39	46.4±4.02	0.47±0.10	2.0±0.10
T16D	-	-	-	-	-	-	-	-	-	-
Q63E	10.58±0.39	6.53±0.24	0.10±0.01	65.3	1.0±0.05	23.57±1.72	14.5±0.84	76.45±3.67	0.19±1.39	2.3±0.16
T95E	-	-	-	-	-	-	-	-	-	-
M121C/T169M	22.6±0.28	13.95±0.17	0.23±0.007	60.73	1.5±0.09	-	-	-	-	-
M121C	8.23±0.13	4.83±0.08	0.25±0.02	30.20	1.7±0.21	-	-	-	-	-
T169M	11.99±0.36	7.40±0.22	0.25±0.02	29.6	1.8±0.20	-	-	-	-	-

Results are means (±SE) for 3 determinations from at least 3 protein preparations.

Only the Q63E mutant preserved the hyperbolic kinetic *vs.* L-ASN of the wild type enzyme; the M121C, M121C/T169M and T169M mutants showed a slightly cooperative kinetic toward L-ASN (n_H_, 1.5, 1.7 and 1.8, respectively).

The double mutant was the less affected in catalytic activity (k_cat_, nearly 70% with respect to that of the wild type) and its catalytic efficiency was even comparable to that of the wild type enzyme (k_cat_/S_0.5_, 60.73 s^-1^ mM^-1^ *vs* 66.4 s^-1^ mM^-1^, respectively), owing to a slightly higher affinity toward L-ASN (S_0.5_ for M121C/T169M: 0.23 *vs* wt: 0.29 mM). The M121C and T169M mutants had reduced catalytic activities (nearly 2.5- to 4-fold *vs* wild type enzyme), but the Q63E mutant, in spite of its 3-fold lower catalytic rate (k_cat_ for Q63E: 6.53 sec^-1^ *vs* wild type: 19.26 sec^-1^), displayed a catalytic efficiency superimposable to that of the wild type enzyme, thanks to its 3-fold higher affinity (S_0.5_ for Q63E: 0.10 mM *vs* wild type: 0.29 mM).

As for L-GLN, only the Q63E mutant showed the capability to hydrolyze this substrate. Moreover, it had a cooperative kinetic like the wild type enzyme (n_H_, 2.3 *vs.* 2.0 of the wild type, Fig. 2B and Table 3), though with a 2.5 times reduced catalytic efficiency.

### Protein stability

**Figure 3:**
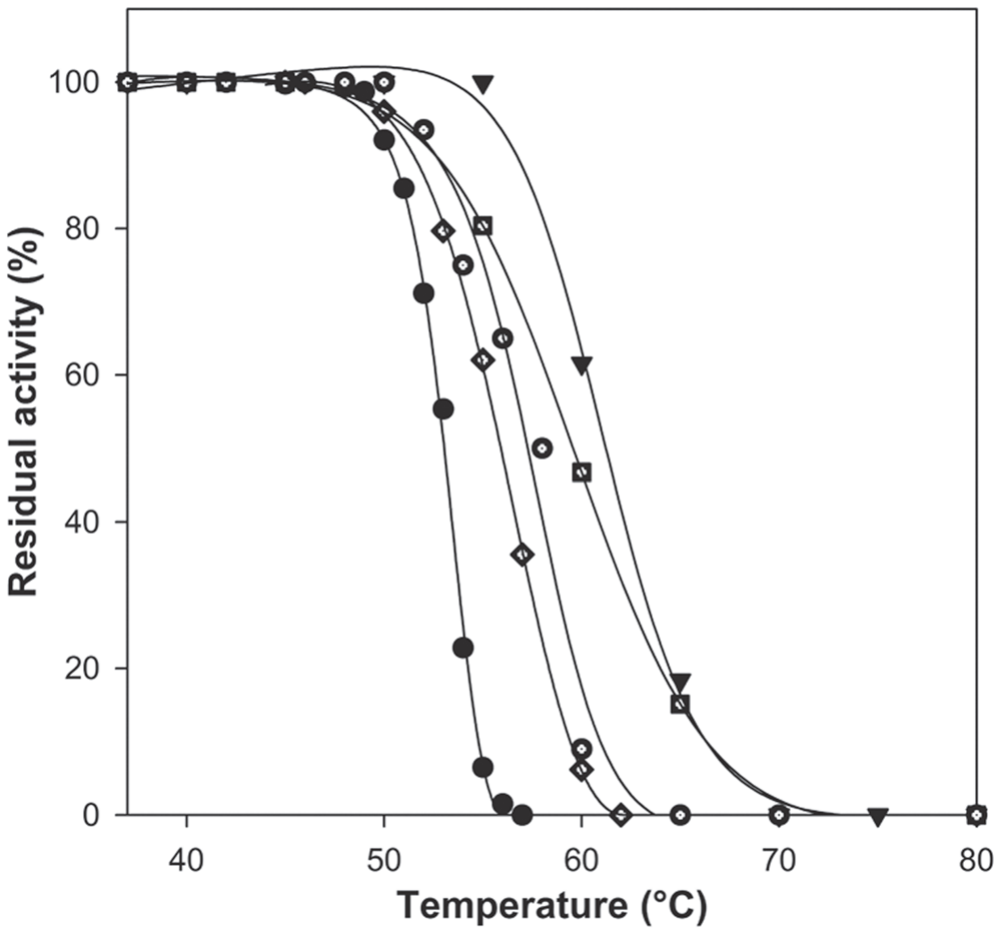
Protein stability of recombinant HpASNase wild type and variants. Plots of the residual activities at 10 min incubation *vs.* temperature. The enzyme solutions were subjected to heat inactivation in a given range of temperatures. Residual activities are expressed as a percentage of initial activity. Black circle: wild type; diamond: Q63E; empty square: M121C; empty circle: T169M; black triangle pointing down: M121C/T169M (double mutant).

Protein stability was evaluated incubating the variants in a wide range of temperatures (40–70°C) within 10 min and calculating T_50_ values after evaluation of their residual activities, as reported in the Materials and Methods section. All the enzymes endowed with L-ASNase activity resulted to be very thermostable (Fig. 3) with T_50_ values 3–8°C higher than the wild type enzyme (Table 4). Particularly, the M121C/T169M double mutant showed a T_50_ value 7°C higher than the wild type (61°C *vs*. 53°C, respectively). This high thermostability would be helpful for both industrial and therapeutic purposes.

**Table 4:** Thermal stability of recombinant HpASNase wild type and variants.

	**T_50_ (°C)**
wild type	53
T16D	-
Q63E	56
T95E	-
M121C/T169M	61
M121C	60
T169M	58

### L-asparaginases cytotoxicity

**Figure 4:**
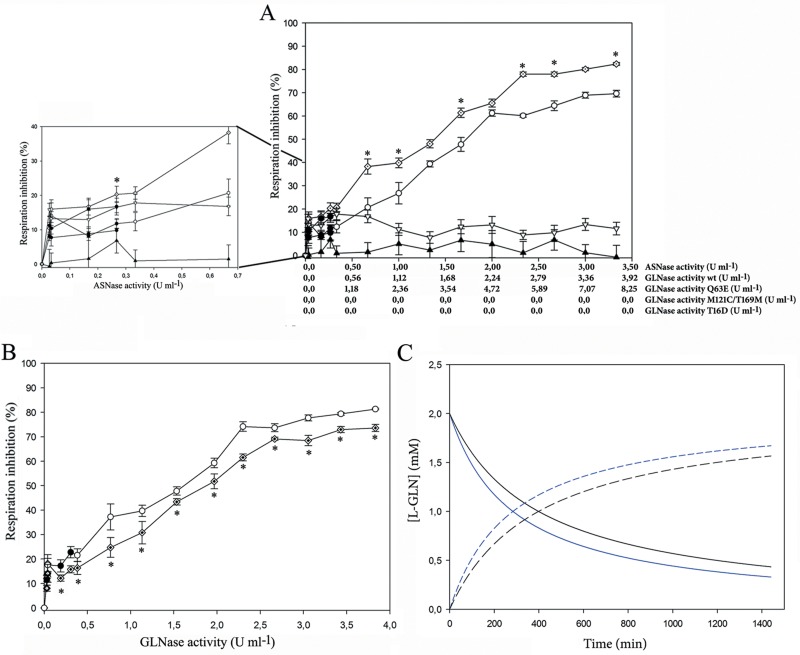
Cell respiration inhibition on HL-60 by wild type HpASNase and its variants as a function of different enzyme units. Panel A: correlation between asparaginase activity and cell respiration inhibition. Inset: low asparaginase activity concentration respiration inhibition. Panel B: correlation between glutaminase activity and cell respiration inhibition. Symbols: black circle: wild type; black triangle pointing up: T16D; diamond: Q63E; black triangle pointing down: M121C/T169M (double mutant). Empty symbols indicate significance *vs* the negative control (p<0.05). * indicate significance (p<0.05) between values for wild type and Q63E mutant. Panel C: L-glutamine consumption analysis for the wild type (straight black line) and Q63E mutant (straight blue line) using U of glutaminase activity (mmol l^-1^ min^-1^) corresponding to the protein amount used for analysis at 3.3 U ml^-1^ of asparaginase activity. Ammonia production is as well reported in the figure (wild type: dashed black line; Q63E: dashed blue line). Analysis was performed using the COPASI 4.12 software. The time course analysis was done according to the Hill cooperativity function and using the following kinetic parameters: wild type: rate 3.81 mmol l^-1^ min^-1^, S_0.5_ 46.4 mM, n_H_ 2; Q63E: rate 7.35 mmol l^-1^ min^-1^, S_0.5_ 76.45 mM, n_H_ 2.3. Initial glutamine concentration: 2 mM.

The cytotoxic effect of variable concentrations of the wild type, T16D, Q63E and M121C/T169M L-asparaginases on the human promyeloid HL-60 cells is reported in Fig. 4A and B and Table 4. For the mutant enzymes, the protein dosage was scaled to match the wild type activity (U ml^-1^) both toward L-ASN and L-GLN. In the case of the inactive T16D mutant, the amount of protein as mg ml^-1^ was considered. Mutants M121C and T169M were not tested on cells because they showed no GLNase activity and half the ASNase catalytic efficiency of the wild-type and were therefore considered not very informative.

The wild type enzyme halved the viability of the cells when used at 1.58 U ml^-1^ (Table 5). The T16D enzyme, devoid of any catalytic activity, did not exhibit any cytotoxic effect. The double mutant, devoid of GLNase activity, exhibited a negligible and non-ASNase dose-dependent cytotoxic effect, which was not significant if compared to the inactive T16D mutant (p>0.05). In fact, viability reduction was significant only if compared with the negative control (cells treated in the absence of enzyme, p>0.05). The Q63E mutant, the only enzyme exhibiting both L-ANSase and L-GLNase activities, although partly reduced, showed a cytotoxic effect comparable with or higher than the wild type enzyme if the same concentration (U ml^-1^) of ASNase was used (EC_50_; Q63E:, 1.37 U ml^-1^ *vs* wild type: 1.58 U ml^-1^, Fig. 4A and Table 5). When the treatment was performed with similar U ml^-1^ of GLNase activity, Q63E showed a reduced cytotoxic effect (EC_50_ wt: 1.58 U ml^-1^ and Q63E: 1.73 U ml^-1^, Table 5).

**Table 5:** EC_50_ values for HpASNase wild type and variants (T16D, Q63E, M121C/T169M).

	**EC_50_ (U ml^-1^)**	
	**Asparaginase activity**	**Glutaminase activity**
wild type	1.58	1.58
T16D	ND	ND
Q63E	1.37	1.73
M121C/T169M	ND	ND

ND: value not detectable. In the case of the T16D and M121C/T169M the data points did not fit the four parameter logistic curve.

## Discussion

**Figure 5:**
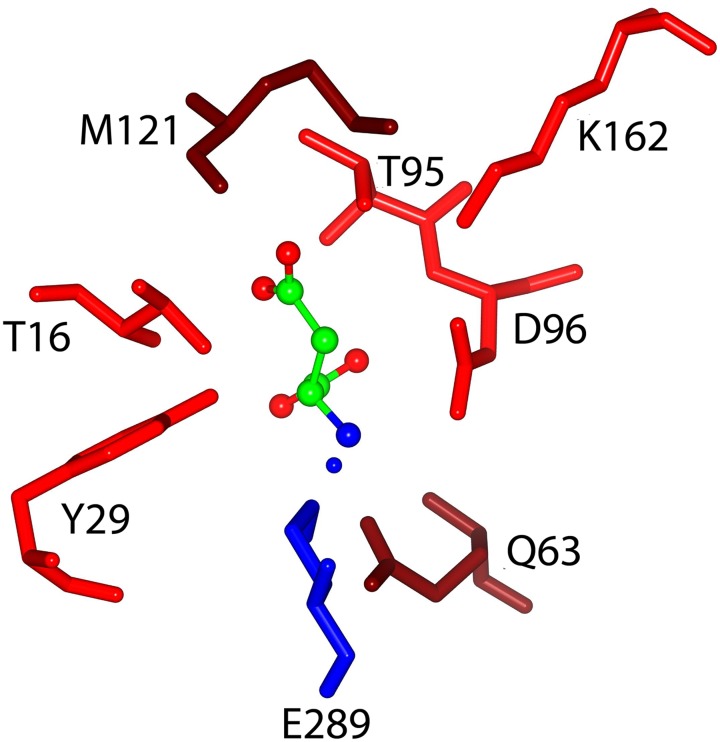
Residues involved in substrate binding/stabilization, and/or catalysis. L-ASN is reported as a ball and stick model. Red/tan residues belong to the A chain, blue residues belong to the C chain. Red and blue residues belong to the catalytic triads. Elements from each subunity of an intimate dimer participate to substrate binding/stabilization and catalysis. Met121 is located nearby the catalytic Thr95 residue and participate to create the hydrophobic environment necessary for Thr95 activation; Gln63 (subunity A) stabilize the catalytic Glu289 (subunity C) of the close monomer.

The rational engineering of six HpASNases here described included two groups of mutants, namely, catalytic triads mutants (T16D, T95E) and substrates binding and stabilization mutants (Q63E, M121C/T169M, M121C, T169M) (Fig. 5).

The T16D and T95E replacements, which have never been experimented before in ASNase, were performed to verify the role of HpASNase threonines in substrate binding and catalysis. L-ASNases carry out a ping-pong like catalysis mechanism that involves transitory and sequential nucleophilic attacks resolved in the release of ammonia and aspartic or glutamic acid. The two threonines of the triads (Thr12 and Thr89 in EcAII and the corresponding Thr16 and Thr95 in HpASNase) and several water molecules are reported to be involved in this mechanism [24].

Crystallographic data show that Thr16 acquires hydrogen bonds through its OG1 atom, exclusively from elements of the side chain of the substrate (CA, CB, CG, OD1, OD2), while the Thr95 OG1 interacts also with the O of the carboxylic group in α of the main chain [25]. We replaced Thr16 with an Asp residue to increase the charge in the active site region maintaining the steric occupancy, thus possibly increasing the affinity of the enzyme for the L-ASN substrate. Replacement of Thr95 with a Glu, which led to the extension of the side chain of the residue, had the purpose of reducing the active site space thus blocking L-GLN accommodation and allowing, at the same time, the interaction of the L-ASN main chain with the Glu carboxylic function. Each replacement on both triad threonines (Thr16 and Thr95), however, resulted to be deleterious for enzyme activity, which is consistent with the characteristics of a previously reported EcAII T89V mutant [30] and with their expected essential role in catalysis [24, 25].

Regarding the second group of mutants, amino acid replacement concerned the Gln63 residue that, with Asn255 and Glu289, is located around the active site and is involved in substrate stabilization and in modulating the accessibility of the binding pocket [25]. Specifically, Gln63 makes hydrogen interactions (OE1) with the α-NH_3_^+^ of the substrate (L-ASN or L-GLN). Its replacement with a Glu leads to the introduction of a carboxylic group at position 63 of the polypeptide chain, resulting in a locally increased negative charge, which should allow a better interaction with L-ASN as a substrate. In fact, the characterization of the Q63E mutant confirmed the residue role in both substrates binding and catalysis. The variant had a catalytic efficiency (k_cat_/S_0.5_: 65.3 s^-1^ mM^-1^) *vs* L-ASN similar to the wt enzyme (k_cat_/S_0.5_, 66.4 s^-1^ mM^-1^), with a reduction of catalytic activity (nearly 3-fold), but increased affinity (nearly 3-fold), and halved glutaminase efficiency (k_cat_/S_0.5_: 0.19 *vs* 0.47 s^-1^ mM^-1^) due to the reduction of both catalytic activity and affinity. Several mutants on the corresponding EcAII residue (Q59) have been characterized to have perturbed ASNase and GLNase activities [16, 19].

In order to discuss the M121C mutant, we need to consider that, beside the flexible loop of the active site (HpASNase residues 19–46), at least two other highly conserved loops play a key role in active site stabilization: a left-handed crossover β4-β5 loop (HpASNase residues 119–153) and the active site forming β13–14 loop (HpASNase residues 286–297) [25]. Residue M121 is highly conserved in the structure of over 500 bacterial L-ASNases [31] and plays two main roles: stabilization of the left-handed cross-over loop on the active site and creation of a hydrophobic environment for the stabilization of catalytic Thr95. M121 is one of the few residues that differentially interacts with L-ASN and L-GLN [32]. Indeed, it creates hydrogen bonds with the L-GLN NE through the sulphur atom of its side chain but does not interact with L-ASN. The M121C replacement introduces a shortened hydrophilic residue that should lose the capability of interacting with L-GLN, leaving the affinity *vs* L-ASN unchanged.

During the mutagenesis experiments to produce the M121C mutant, an unwanted, additional random mutation was introduced, leading to the formation of the M121C/T169M mutant, namely a double mutant. Very little is known about the Thr169 residue, though the corresponding EcAII residue (Thr163) is reported to be involved in intersubunits interactions [4, 23]. To further investigate the role of T169, we also built the single T169M mutant. In the structure of the HpASNase monomer, Thr169 is involved in hydrogen bonds with the O of the Thr176 and with two water molecules. None of these structural elements seem to be directly involved in enzyme catalysis.

Replacements of either Met121, or Thr169, or both (Met121/Thr169) killed the enzyme GLNase activity leaving the affinity toward L-ASN unchanged. Interestingly, in all the three cases mutants displayed positive cooperativity *vs* L-ASN, underlining alterations within enzyme subunits interactions.

**Figure 6:**
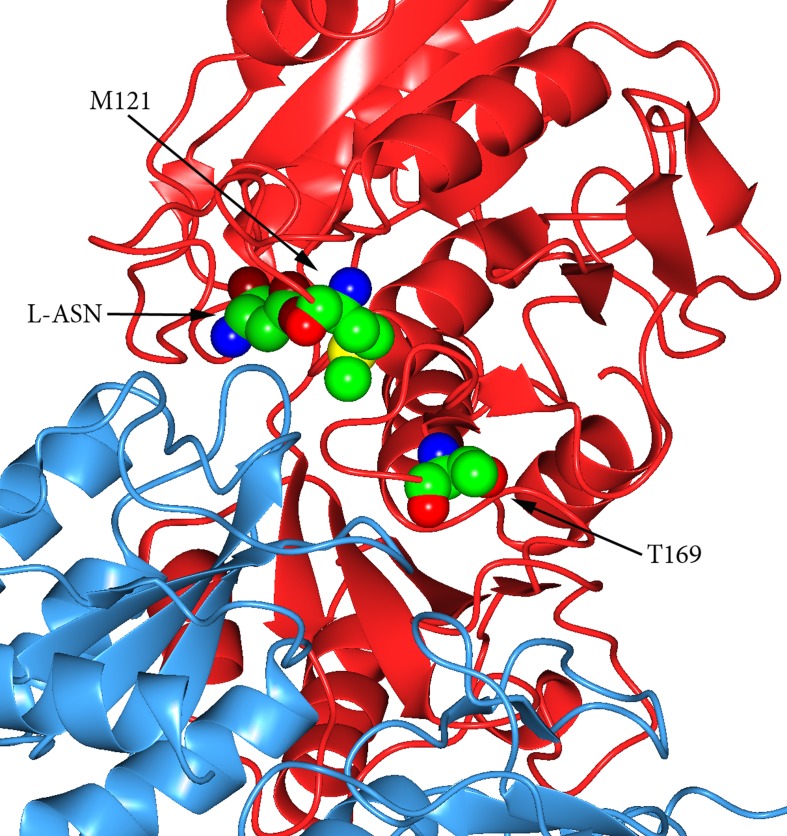
Residue M121 and T169 localization. L-ASN, Met121 and Thr169 are represented as spheres; the subunits A (red) and C (light blue) are represented as ribbons. Thr169 locates in a loop shared by intersubunits regions and, in *E. coli*, it resulted to interact with residues of the near subunit in the intimate dimer. Residue Met121 locates far from the Thr169 residue. Both residues do not seem to interact with each other.

Both mutants carrying each single mutation (M121C or T169M) had a reduced ASNase activity, but the double mutant had an activity quite similar to the wild type enzyme. Its preserved activity toward L-ASN most likely depends on a complementary action of the two mutations affecting residues located in two critical regions of the enzyme structure: residue Met121, near the active site region, and Thr169, in the intersubunit one (Fig. 6). The distance of Thr169 from the active site suggests the possibility to interfere with the enzyme activity even tackling regions seemingly unrelated to the catalytic mechanism.

The cytotoxic effect of the T16D, Q63E and M121C/T169M mutants was assayed on HL-60 cells. The inactive T16D mutant did not exert any cytotoxic effect, proving that the anti-proliferative efficacy of HpASNase is related only to its enzymatic activity and is independent from unknown features of the protein or from the recombinant nature of the enzyme carrying the N-terminal His-tag. The double mutant, completely devoid of GLNase activity, had a very low cytotoxic effect that was not dependent on the enzyme dose, an effect confirmed by Trypan blue exclusion in slightly different conditions [33].

Previous work on EcAII [16] has shown that GLNase activity is essential for cell cytotoxicity only when ASNS is expressed. In the model proposed by the Authors, L-GLN, one of the substrates of ASNS, is removed by the secondary GLNase activity of ASNase, thus contributing to enhance cytotoxicity. HL-60 cells are known to express ASNS [34], thus explaining the effects we observed at comparable units of ASNase. In the absence of GLNase activity (M121C/T169M mutant), the effect on cell viability was similar to the one exerted by the T16D mutant, devoid of any activity. In the presence of intermediate GLNase units (wild-type), cytotoxicity became relevant. It is interesting to note that, doubling GLNase units (Q63E mutant) compared to the wild-type, the further gain in cytotoxicity is only about 10% and this, because of the subsaturating L-GLN concentrations in which the experiments were performed (2 mM, as initially added to the culture medium), is probably related not to the lower affinity of Q63E for L-GLN with respect to the wild-type, but to its roughly halved GLNase efficiency (k_cat_/S_0.5_ *vs* L-GLN, wt: 0.47 and Q63E, 0.19). In fact, mathematical modelling by COPASI of the relative GLNase activity for the wild type and Q63E mutant, respectively, at similar ASNase units (Fig. 4C) shows both a slightly quicker consumption of L-GLN and accumulation of ammonium in the mutant.

In EcAII mutants [16], affinity for GLNase has been engineered to remove its side effects and this is particularly relevant for ASNS negative leukemia clones. On the other hand, for leukemia clones expressing ASNS, modulation of GLNase catalytic efficiency could be relevant to optimize its therapeutic index.

All in all, the data obtained from our cytotoxicity tests revealed that GLNase activity is absolutely needed for the HpASNase anti-proliferative effect on HL-60 cells and that its modulation can contribute to reach the enzyme maximal cytotoxic effect.

## Conclusions

The biochemical characterization of the six variants of HpASNase, whose proper folding and quaternary structure were systematically verified, showed that substitution of the catalytic triads residues completely impairs the enzyme capability to carry out the reaction, underlining their essential role. On the other hand, substitutions in the region of substrates binding and stabilization resulted in a reduction of the catalytic rate toward L-ASN in all cases except one (M121C/T169M), and in a reduction (Q63E) or complete removal of the glutaminolytic activity, indicating that co-participation of other structural elements besides the triads elements is relevant for the catalytic cycle.

The production of ASNase variants with reduced or null glutaminolytic activity has allowed to verify the importance of GLNase activity in the cytotoxicity exterted on HL-60 cells, confirming that modulation of the GLNase kinetic parameters of ASNase is a relevant issue to consider in order to optimize the outcome of its therapeutic applications.

## Supporting Information

S1 Fig Wild type and T16D, Q63E, T95E, M121C, T169M, M121C/T169M mutants amino acids alignment. The amino acids sequences derived from each mutant nucleotide sequencing are aligned to the one of the wild type enzyme. The wild type derived secondary structure is as well reported. α-helices, 3 -helices and π-helices are displayed as medium, small and large squiggles respectively. β-strands are rendered as arrows, strict β-turns as TT letters and strict α-turns as TTT. (ESPript -http://espript.ibcp.fr). Robert, X. and Gouet, P. (2014) “Deciphering key features in protein structures with the new ENDscript server”. Nucl. Acids Res. 42(W1), W320-W324. doi: 10.1093/nar/gku316 (freely accessible online).
(TIF)
