# Supplementary figures and images for: Engineering of Helicobacter pylori L-Asparaginase: Characterization of Two Functionally Distinct Groups of Mutants
Source: PLoS One. 2015 Feb 9;10(2):e0117025. doi: 10.1371/journal.pone.0117025 (PMC4321988; doi:10.1371/journal.pone.0117025)

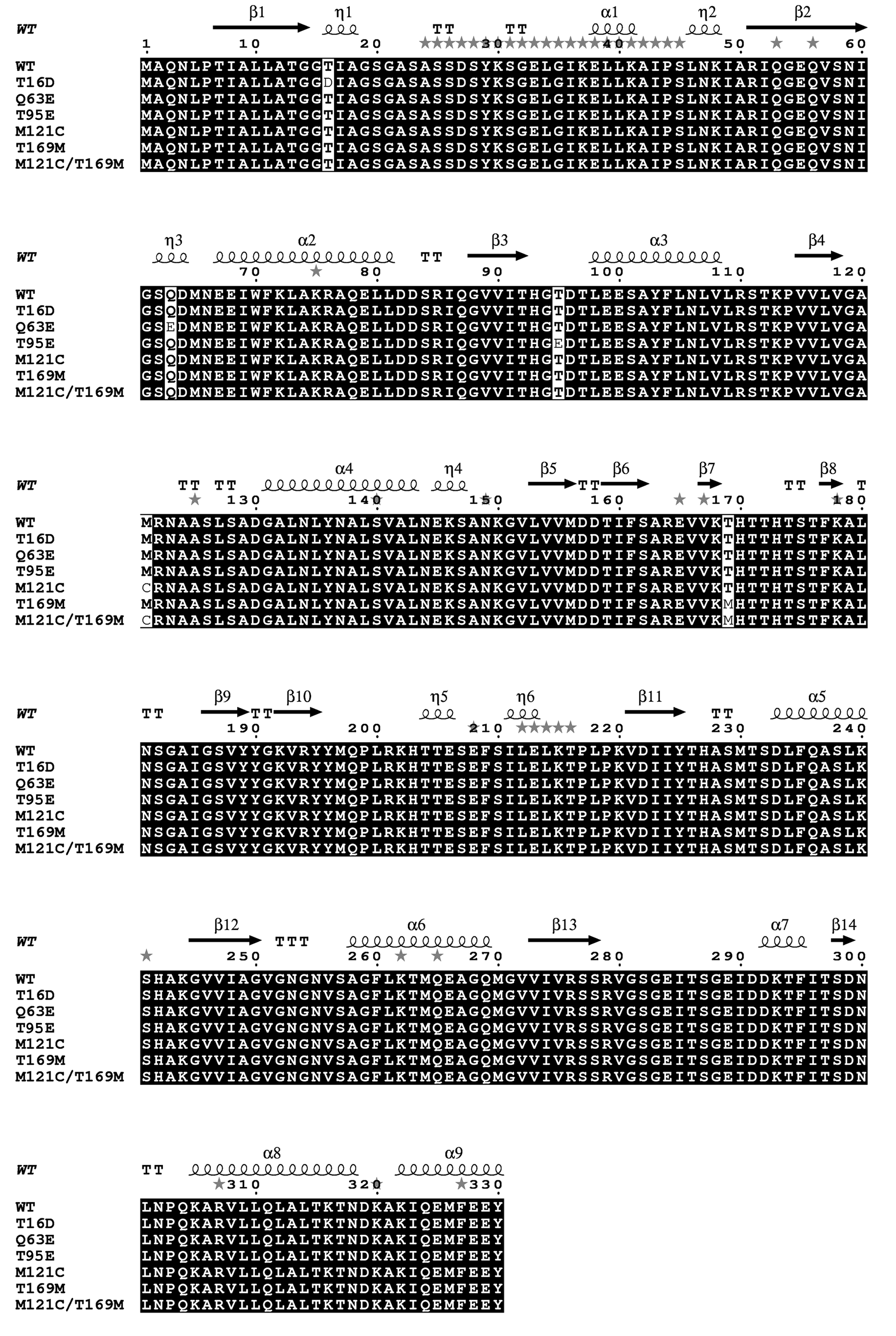

Supplement: S1 Fig — The amino acids sequences derived from each mutant nucleotide sequencing are aligned to the one of the wild type enzyme. The wild type derived secondary structure is as well reported. α-helices, 3 -helices and π-helices are displayed as medium, small and large squiggles respectively. β-strands are rendered as arrows, strict β-turns as TT letters and strict α-turns as TTT. (ESPript -http://espript.ibcp.fr). Robert, X. and Gouet, P. (2014) “Deciphering key features in protein structures with the new ENDscript server”. Nucl. Acids Res. 42(W1), W320-W324. doi: 10.1093/nar/gku316 (freely accessible online). (TIF) [file pone.0117025.s001.tif]
